# Clinical Factors Associated with Pain During Outpatient Hysteroscopy in a Standardized Vaginoscopic Setting: A Prospective Observational Study

**DOI:** 10.3390/jcm15166335

**Published:** 2026-08-16

**Authors:** Francesco Pio Toscano, Maria D’Angelo, Luigi Riello, Mario Ardovino, Antonio Mollo

**Affiliations:** 1 Department of Public Health, Faculty of Medicine, University of Naples Federico II, 80131 Naples, Italy; maria.dangelo@aornmoscati.it (M.D.); rielloluigi8@gmail.com (L.R.); 2 Department of Obstetrics and Gynecology, Azienda Ospedaliera San Giuseppe Moscati, 83100 Avellino, Italy; mario.ardovino@aornmoscati.it; 3 Unit of Obstetrics and Gynecology, Clinica Villa del Sole, 81100 Caserta, Italy; 4 Department of Medicine, Salerno Medical School, University of Salerno, 84081 Baronissi, Italy

**Keywords:** office hysteroscopy, outpatient hysteroscopy, pain perception, cervical access, patient tolerability, see-and-treat, prospective observational study

## Abstract

**Background:** Outpatient hysteroscopy is the reference standard for the diagnosis and treatment of benign intrauterine pathology. Although several pharmacological and non-pharmacological strategies have been proposed to reduce procedural pain, the clinical and procedural factors primarily determining patient tolerability in a standardized vaginoscopic setting remain incompletely understood. This prospective study aimed to identify the main predictors of pain, procedural tolerability, successful completion, and patient acceptance during outpatient hysteroscopy. **Methods:** A prospective observational study was conducted between February and June 2026 at a second-level referral hospital. Consecutive women undergoing outpatient hysteroscopy were enrolled. All procedures were performed according to a standardized vaginoscopic “no-touch” protocol using a 15-Fr Bettocchi hysteroscope. Patients completed structured questionnaires before and after the procedure, while physicians recorded procedural findings immediately afterward. Clinical characteristics, psychological variables, procedural factors, pain scores, procedural tolerability, successful completion, and future patient preference were prospectively analyzed. **Results:** A total of 105 women were included. Diagnostic hysteroscopy was successfully completed in 94 patients (89.5%), and a see-and-treat procedure was performed in 57 (54.3%). Difficult cervical access occurred in 37 cases (35.2%) and emerged as the strongest determinant of poor procedural tolerability. These patients experienced significantly higher maximum pain scores (7.35 ± 2.81 vs. 4.04 ± 2.51; *p* < 0.001), a higher prevalence of severe pain (67.6% vs. 20.6%; *p* < 0.001), poorer procedural tolerability, greater preference for future hysteroscopy under anesthesia, and a higher risk of incomplete diagnostic examination. All incomplete hysteroscopies occurred in patients with difficult cervical access. Expected procedural pain was significantly associated with maximum pain (*p* = 0.0016), whereas pre-procedural anxiety was not. More than half of the patients identified cervical passage as the most painful procedural step. Patients undergoing successful see-and-treat procedures reported the lowest pain scores, suggesting that the operative phase itself was not the principal determinant of procedural discomfort. **Conclusions:** In a standardized vaginoscopic setting, procedural tolerability during outpatient hysteroscopy appears to depend primarily on difficult cervical access rather than on the operative intervention itself. Pain expectation may influence the hysteroscopic experience more than pre-procedural anxiety, whereas successful see-and-treat procedures remain well tolerated once uncomplicated access to the uterine cavity has been achieved. Early identification of patients at increased risk of difficult cervical access may improve patient counseling, optimize procedural completion, and preserve the benefits of outpatient hysteroscopy.

## 1. Introduction

Hysteroscopy is the gold standard for the evaluation of intrauterine pathology in both premenopausal and postmenopausal women. It has been described as a “Copernican revolution” in the diagnosis and treatment of uterine pathology because of its profound impact on modern gynecologic practice [1].

Operative hysteroscopy represents an effective uterus-preserving treatment for several benign intrauterine conditions and is among the most commonly used procedural alternatives to hysterectomy [2].

Hysteroscopy can be performed either on an outpatient basis or as an inpatient procedure under anesthesia, and both examination modes have been shown to be equally effective [3,4].

Technological advances, including the miniaturization of hysteroscopes and the introduction of 5-Fr operative instruments, have progressively expanded its therapeutic potential, leading to the development of the “see-and-treat” approach [1]. This concept integrates diagnosis and treatment into a single procedure, allowing both to be performed during the same outpatient visit [1].

Outpatient hysteroscopy has been associated with higher levels of pre-procedural anxiety than hysteroscopy performed under general anesthesia [5,6,7,8]. However, whether anxiety itself contributes to procedural pain remains controversial, with conflicting results reported in the literature.

In Italy, many gynecologic procedures are still routinely performed in the operating room, particularly in first-level hospitals, while university and second- or third-level referral centers have increasingly implemented outpatient approaches. This highlights the need for more robust evidence regarding the effectiveness, feasibility, and patient acceptability of outpatient hysteroscopy.

Pain is the primary reason for abandonment of the procedure or incomplete assessment [9] and pain experienced during outpatient hysteroscopy may compromise patient cooperation, reduce the likelihood of successful procedure completion, negatively affect the overall patient experience, and influence the willingness to undergo future hysteroscopic procedures [5].

Despite the widespread adoption of outpatient hysteroscopy, patient-reported pain remains one of the main factors limiting its acceptance and successful completion. Previous studies have identified several potential determinants of procedural pain, including menopausal status, cervical stenosis, parity, previous cervical procedures, anxiety, and operator experience; however, available evidence remains heterogeneous and often derives from studies performed using different hysteroscopic techniques, instrumentation, analgesic protocols, and procedural settings [9,10,11,12,13]. Consequently, it remains unclear which clinical and procedural factors most strongly influence pain perception, procedural tolerability, successful completion of the examination, and patient acceptance in the outpatient hysteroscopy setting.

Therefore, the aim of this prospective observational study was to investigate the clinical and procedural factors associated with pain and patient tolerability during outpatient hysteroscopy performed in a standardized vaginoscopic setting. Secondary objectives were to evaluate the association between pain, procedural completion, patient-reported tolerability, and future patient preference.

## 2. Materials and Methods

### 2.1. Study Design, Setting and Hysteroscopic Procedure

A prospective observational study was conducted between February and June 2026 at a second-level referral hospital in Southern Italy. Consecutive women scheduled for outpatient hysteroscopy during the study period were invited to participate. Patients were enrolled irrespective of the indication for hysteroscopy. Referral indications reflected routine clinical practice and could have been established either within our institution or by external gynecologists. No exclusion criteria were applied; the only reason for non-inclusion was refusal to complete the study questionnaire.

**Figure 1:**
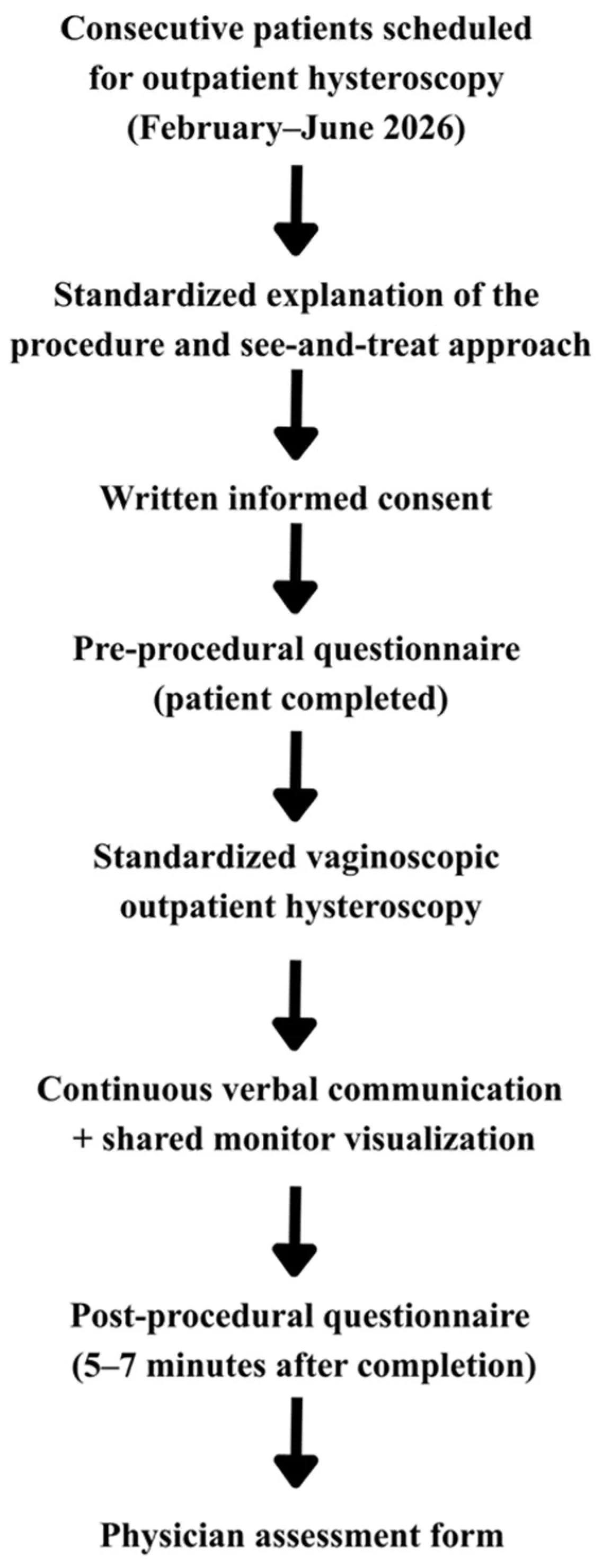
Flowchart of the standardized study protocol and data collection process.

Before the procedure, all patients received a standardized explanation of outpatient hysteroscopy and were informed that, if an intrauterine pathology suitable for immediate treatment was identified, an operative procedure could be performed during the same session according to the “see-and-treat” approach. After written informed consent was obtained, all participants completed the pre-procedural questionnaire. The overall study workflow and data collection process are summarized in Figure 1.

All hysteroscopic procedures were performed according to a predefined standardized protocol to minimize procedural variability and reduce potential sources of bias in pain assessment.

A vaginoscopic “no-touch” approach was used in all cases with a 15-Fr Bettocchi office hysteroscope (Karl Storz, Tuttlingen, Germany) equipped with a 30° rod-lens optic and an oval-profile continuous-flow sheath. The oval-profile design is intended to better conform to the natural anatomy of the cervical canal, while the 30° optic allows visualization of the uterine cavity through rotation of the hysteroscope rather than lateral displacement of the shaft, thereby minimizing cervical manipulation [14]. The uterine cavity was distended with normal saline delivered using a pressure-controlled HAMOU^®^ ENDOMAT^®^ irrigation pump (Karl Storz, Tuttlingen, Germany), maintaining an intrauterine pressure between 70 and 80 mmHg. Whenever an operative procedure was indicated, only 5-Fr mechanical instruments (grasping forceps and cold scissors) were used, avoiding the routine use of electrosurgical devices. This approach was adopted to standardize the operative technique and because current evidence suggests that mechanical instruments are associated with lower procedural pain than electrosurgical devices during outpatient hysteroscopy [15].

Operative procedures were performed according to the principles of the “see-and-treat” approach and included endometrial biopsy, targeted biopsy, endometrial polypectomy, cervical polypectomy, intrauterine device removal, hysteroscopic metroplasty, and other minor procedures when clinically indicated. More than one operative procedure could be performed during the same hysteroscopic session when required.

Of the 105 hysteroscopies included in the study, 77 were performed by the same operator, whereas the remaining procedures were carried out by other gynecologists within the unit. However, the principal operator was present during all procedures to ensure consistent application of the standardized protocol and to minimize inter-operator variability.

### 2.2. Questionnaire and Data Collection

A structured questionnaire was specifically developed for prospective data collection to systematically evaluate patient- and procedure-related factors potentially associated with pain perception, procedural tolerability, successful completion of outpatient hysteroscopy, and patient acceptance of the procedure.

The variables included in the questionnaire were selected based on the main factors previously reported in the literature to be associated with pain perception and tolerability during outpatient hysteroscopy, including obstetric history, menopausal status, previous cervical procedures, chronic pelvic pain, anxiety, and procedure-related factors [16,17]. The selection of these variables was also informed by previous prospective observational studies that systematically evaluated patient characteristics and procedural factors as potential predictors of pain during office hysteroscopy [18].

In addition, further questions were developed by the authors based on clinical experience and the objectives of the present study to investigate aspects that have been only sparsely explored in the literature, including expected procedural pain, the hysteroscopic phase perceived as the most painful, patient preference regarding a future outpatient or inpatient hysteroscopy, and technical factors potentially associated with unsuccessful procedure completion. The questionnaire was specifically developed to provide a multidimensional assessment of the outpatient hysteroscopy experience by integrating patient-reported outcomes, procedural characteristics, and operator-reported findings collected at predefined time points [18].

The questionnaire consisted of three separate sections completed at predefined time points throughout the patient pathway (Supplementary Material File S1).

The first section was completed by the patient immediately before hysteroscopy and collected baseline clinical characteristics, obstetric and gynecological history, previous cervical or hysteroscopic procedures, the presence of chronic pelvic pain or dyspareunia, pre-procedural anxiety, and expected procedural pain.

The second section was completed by the patient approximately 5–7 min after completion of the procedure, once she had dressed and before leaving the outpatient clinic. This section recorded the maximum pain experienced during hysteroscopy using an 11-point Numeric Rating Scale (NRS; 0 = no pain, 10 = worst imaginable pain), the procedural phase perceived as the most painful, residual pain immediately after the examination, overall procedural tolerability, and the patient’s preference regarding a future hysteroscopy performed either in an outpatient setting or under anesthesia.

The third section was completed immediately after the procedure by the attending physician and recorded the main procedural characteristics, including successful completion of the diagnostic examination, performance and type of operative procedure, unsuccessful completion of the see-and-treat procedure due to pain, difficult cervical access, uterine position, and immediate procedural complications, including vasovagal reactions.

For the purposes of the present study, an operative procedure was defined as a hysteroscopic intervention successfully performed after completion of the diagnostic assessment. Conversely, failed see-and-treat procedures were defined as cases in which the diagnostic hysteroscopy was successfully completed and an operative indication was identified, but the planned operative treatment could not be performed because of poor patient tolerability or inability to proceed with the operative step.

The division of the questionnaire into three sections, completed at different time points and by different respondents, was designed to enable prospective and standardized collection of both patient-reported outcomes and procedure-related data, thereby minimizing recall bias and providing a comprehensive assessment of the hysteroscopic experience. All study variables were prospectively collected according to a predefined protocol before statistical analysis.

Throughout all procedures, continuous communication with the patient was maintained. The operator progressively explained each procedural step (vaginal access, cervical passage, uterine cavity inspection, and, when applicable, the operative phase), while encouraging the patient to observe the hysteroscopic images on the monitor. In addition, a strategy of continuous verbal support based on reassurance and conversational distraction (“verbal anesthesia”) was routinely adopted throughout the procedure to reduce anxiety, promote patient cooperation, and improve procedural comfort.

Cervical access was classified as “difficult” whenever entry into the uterine cavity required additional maneuvers beyond the standard vaginoscopic technique. Specifically, difficult cervical access was defined by the need to perform blunt adhesiolysis using the tip of the hysteroscope, the use of miniaturized 5-Fr mechanical instruments to facilitate passage through the cervical canal, the presence of stenosis involving the external cervical os, the cervical canal, or the internal cervical os, as well as marked cervical tortuosity or cervical adhesions hindering hysteroscopic progression. This operational definition was developed based on the principal descriptions available in the literature regarding anatomical obstacles to hysteroscopic access and the hysteroscopic techniques employed to overcome them [13,19]. To minimize inter-operator variability in the identification of difficult cervical access, most procedures (77/105) were performed by the same operator. For the remaining procedures, the same operator was present throughout the examination, ensuring consistent application of the predefined classification criteria and the standardized hysteroscopic protocol.

### 2.3. Statistical Analysis

Data were extracted from the original Microsoft Excel database and underwent quality control before statistical analysis to verify data completeness, consistency, and coding accuracy. Categorical variables are presented as frequencies and percentages, whereas continuous variables are presented as mean ± standard deviation (SD) and median with interquartile range (IQR). Comparisons between two independent groups were performed using the Mann–Whitney U test, whereas comparisons among more than two groups were performed using the Kruskal–Wallis test, followed, when appropriate, by pairwise post hoc comparisons using the Mann–Whitney U test. Associations between categorical variables were evaluated using the chi-square test or Fisher’s exact test, as appropriate. Correlations between continuous or ordinal variables were assessed using Spearman’s rank correlation coefficient (ρ). Odds ratios (ORs) with 95% confidence intervals (95% CIs) were calculated, where appropriate, for binary outcomes. Statistical analyses were performed using Python (version is 3.11, Python Software Foundation, Wilmington, DE, USA) with the pandas, SciPy, and statsmodels libraries. All statistical tests were two-sided, and a *p*-value < 0.05 was considered statistically significant.

## 3. Results

A total of 105 women undergoing outpatient hysteroscopy were prospectively included in the study. Baseline demographic, clinical, and procedural characteristics are summarized in Table 1. The mean age was 50.6 ± 11.5 years, and 52 patients (49.5%) were postmenopausal. Difficult cervical access was encountered in 37 procedures (35.2%), whereas the diagnostic hysteroscopy was successfully completed in 94 patients (89.5%). An operative see-and-treat procedure was performed in 57 cases (54.3%). The mean maximum procedural pain score was 5.2 ± 3.0, while the mean post-procedural pain score was 2.2 ± 2.6.

Patients with difficult cervical access reported significantly higher maximum procedural pain scores than those without difficult cervical access (mean NRS 7.35 ± 2.81 vs. 4.04 ± 2.51; *p* < 0.001) (Table 2). The mean difference in maximum pain score between the two groups was 3.31 NRS points. Severe procedural pain (NRS ≥ 7) was also significantly more frequent among patients with difficult cervical access than among those without difficult cervical access (67.6% vs. 20.6%; *p* < 0.001). Patients with difficult cervical access had higher odds of severe procedural pain compared with those without difficult cervical access (OR 8.04, 95% CI 3.25–19.87) (Table 2).

The cervical passage was identified as the most painful phase of the procedure by 57 patients (54.3%), followed by operative maneuvers in 26 cases (24.8%), uterine cavity distension in 10 cases (9.5%), and uniform pain or inability to identify a specific painful phase in 12 cases (11.4%) (Table 3). Pain intensity differed significantly according to the phase identified as the most painful (*p* < 0.001). Patients reporting cervical passage as the most painful step experienced the highest maximum pain scores (mean NRS 6.44 ± 2.73), whereas patients identifying operative maneuvers as the most painful phase reported lower pain scores (mean NRS 3.69 ± 2.53) than those identifying cervical passage or uterine cavity distension. The lowest pain scores were observed in patients reporting uniform pain or being unable to identify a specific painful phase (mean NRS 2.08 ± 2.19). Post hoc analysis showed a significant difference between the cervical passage and operative maneuvers groups (Mann–Whitney U = 1145, *p* = 6.45 × 10^−5^).

A significant positive correlation was observed between expected pain before the procedure and maximum procedural pain (*p* = 0.0016) (Table 4). In contrast, pre-procedural anxiety was not significantly correlated with maximum procedural pain (*p* = 0.178) (Table 4). Postmenopausal patients reported significantly higher maximum pain scores than premenopausal patients (mean NRS 5.90 ± 2.94 vs. 4.53 ± 3.03; *p* = 0.020) (Table 5).

No significant differences in maximum procedural pain were observed according to uterine position or obstetric history. Patients with a retroverted uterus reported maximum pain scores comparable to those without uterine retroversion (mean NRS 5.56 ± 3.32 vs. 4.61 ± 2.75; *p* = 0.426). Similarly, no significant differences were observed according to previous cesarean section (mean NRS 5.44 ± 3.23 vs. 5.11 ± 2.98; *p* = 0.671) or history of vaginal delivery (mean NRS 5.06 ± 3.33 vs. 5.37 ± 2.76; *p* = 0.551). When patients were further stratified into four obstetric history subgroups, no significant differences in maximum procedural pain were observed (Kruskal–Wallis H = 0.40, *p* = 0.941). Mean maximum pain scores were 5.27 ± 2.68 in nulliparous women (*n* = 26), 5.46 ± 2.89 in women with previous cesarean section only (*n* = 26), 5.02 ± 3.17 in women with previous vaginal delivery only (*n* = 47), and 5.33 ± 4.80 in women with both previous vaginal delivery and cesarean section (*n* = 6).

Maximum procedural pain differed significantly according to operative procedure status (*p* = 0.003) (Table 6). Post hoc analysis showed significantly lower pain scores in patients with successful see-and-treat compared with both failed see-and-treat procedures (*p* = 0.0017) and diagnostic hysteroscopy only (*p* = 0.0420), whereas no significant difference was observed between failed see-and-treat procedures and diagnostic hysteroscopy only (*p* = 0.333). The lowest pain scores were observed in patients who underwent a successful see-and-treat procedure (mean NRS 3.91 ± 2.44), whereas the highest pain scores were observed in patients in whom the see-and-treat procedure could not be completed (mean NRS 6.47 ± 2.85). Patients undergoing diagnostic hysteroscopy without indication for an operative procedure had intermediate pain scores (mean NRS 5.45 ± 2.96).

Poor procedural tolerability was significantly associated with both severe procedural pain (NRS ≥ 7) and difficult cervical access (both *p* < 0.001). Likewise, patients reporting severe procedural pain and those with difficult cervical access were significantly more likely to prefer a future hysteroscopy under anesthesia (both *p* < 0.001). In addition, patients who preferred a future hysteroscopy under anesthesia reported significantly higher expected pain scores before the procedure than those willing to undergo outpatient hysteroscopy again (mean expected pain NRS 7.22 ± 2.49 vs. 6.00 ± 2.63; *p* = 0.043).

Overall, 82 of 105 patients (78.1%) rated the procedure as well tolerated or tolerable, and 82 of 105 (78.1%) stated that they would choose outpatient hysteroscopy again if a repeat procedure were required.

Diagnostic hysteroscopy could not be completed in 11 patients (10.5%). All incomplete procedures occurred in patients with difficult cervical access, and all of these patients reported severe procedural pain (NRS ≥ 7). Both difficult cervical access and severe procedural pain were significantly associated with failure to complete the diagnostic examination (both *p* < 0.001) (Table 7).

## 4. Discussion

Office hysteroscopy is currently the reference technique for the diagnosis and treatment of benign intrauterine pathology owing to its high diagnostic accuracy, the possibility of performing “see-and-treat” procedures, and its overall good patient tolerability. Nevertheless, despite continuous technological advances, the introduction of miniaturized instruments, and the widespread adoption of the vaginoscopic approach, a substantial proportion of women still experience significant pain or poor procedural tolerability, leading to premature interruption of the examination or the need for hysteroscopy under anesthesia. Consequently, over the last two decades, considerable research has focused on developing strategies to improve the tolerability of office hysteroscopy. A wide range of pharmacological and non-pharmacological interventions have been investigated, including the vaginoscopic technique, different local anesthetic approaches, pharmacological cervical priming, alternative uterine distension media, and, more recently, adjunctive interventions such as music therapy, virtual reality, and transcutaneous electrical nerve stimulation [20,21,22,23,24,25,26,27,28]. Although many of these strategies have demonstrated variable degrees of benefit, their primary objective has largely been to reduce procedural pain. By contrast, comparatively less attention has been devoted to identifying the clinical and procedural factors that, within a standardized hysteroscopic setting, primarily influence procedural tolerability and the overall patient experience. In this context, the present study was designed to simultaneously evaluate a broad range of clinical and procedural variables in order to identify the principal determinants of good or poor tolerability during outpatient hysteroscopy.

Overall, our findings suggest that patient tolerability during outpatient hysteroscopy is determined by the interplay between patient-related clinical characteristics and intrinsic procedural factors. By adopting a highly standardized hysteroscopic protocol, we were able to minimize the influence of technical variables and potential procedure-related confounders, allowing a more accurate evaluation of the association between patients’ clinical characteristics, procedural difficulties encountered during the examination, and overall procedural tolerability. The use of a standardized vaginoscopic approach is particularly relevant when interpreting our findings. By avoiding routine cervical instrumentation, the “no-touch” technique minimizes unnecessary cervical manipulation and has previously been associated with shorter procedural times and lower local anesthetic requirements than the conventional approach [29].

Among the patient-related factors evaluated, one of the most interesting findings of the present study concerns the distinct roles of pre-procedural anxiety and pain expectation. Although anxiety has been widely recognized as a potential determinant of the hysteroscopic experience, it was not significantly associated with procedural pain in our cohort (*p* > 0.05). In contrast, expected pain before the procedure was significantly associated with the maximum procedural pain experienced during outpatient hysteroscopy (*p* < 0.05). Taken together, these findings suggest that anxiety and pain expectation represent two distinct dimensions of the hysteroscopic experience. It is plausible that pain expectation constitutes a more specific psychological construct, directly related to the patient’s anticipation of the procedure, whereas anxiety reflects a more general emotional state that is less closely associated with pain perception during the examination. From a clinical perspective, pre-procedural counseling should not only aim to reduce patients’ anxiety but also to establish realistic expectations regarding outpatient hysteroscopy by clearly explaining the different stages of the procedure, the sensations patients may experience, and the strategies adopted to improve procedural tolerability. This finding may help explain previous observations reported by Akca et al., who demonstrated that video-based pre-procedural education significantly improved patient satisfaction, without reducing procedural pain [27]. Although this interpretation is consistent with our findings, it remains a hypothesis that should be confirmed by prospective studies specifically designed to evaluate the role of pain expectation.

Among the procedural factors evaluated, difficult cervical access emerged as the main determinant of poor tolerability during outpatient hysteroscopy. Patients with difficult cervical access experienced significantly higher procedural pain, a greater frequency of severe pain, poorer procedural tolerability, a stronger preference for future hysteroscopy under anesthesia, and a significantly higher risk of incomplete diagnostic examination. Taken together, these findings suggest that difficult cervical access represents far more than a simple technical challenge, substantially influencing the overall hysteroscopic experience. In our cohort, patients with difficult cervical access reported significantly higher maximum pain scores than those with uncomplicated cervical access (7.35 ± 2.81 vs. 4.04 ± 2.51; *p* < 0.001), corresponding to a mean difference of more than three NRS points. Moreover, more than two-thirds of these patients experienced severe procedural pain (NRS ≥ 7), a significantly higher proportion than that observed among women without difficult cervical access (*p* < 0.001).

Passage through the cervical canal, particularly across the internal cervical os, represents one of the most nociceptive phases of the procedure. Cervical stenosis, tortuosity, or other anatomical conditions requiring additional maneuvers to gain access to the uterine cavity inevitably result in greater cervical manipulation, thereby increasing pain perception. Previous studies have consistently identified cervical stenosis as one of the main determinants of pain during outpatient hysteroscopy and one of the leading causes of procedural failure, while also demonstrating the protective effect of vaginal delivery and cervical patency [12,19,30,31]. Our findings further strengthen this concept. All incomplete diagnostic hysteroscopies occurred exclusively in patients with difficult cervical access, and all of these patients experienced severe procedural pain (NRS ≥ 7). Furthermore, difficult cervical access was significantly associated not only with failure to complete the examination but also with poorer procedural tolerability and a greater preference for future hysteroscopy under anesthesia (all *p* < 0.001).

The difficult cervical access appears to be one of the principal determinants of the overall outpatient hysteroscopy experience, simultaneously influencing pain perception, procedural tolerability, successful completion of the examination, and future patient acceptance of the procedure.

The interpretation of our findings is further supported by the fact that more than half of the patients identified passage through the cervical canal as the most painful phase of outpatient hysteroscopy. In contrast, uterine cavity distension and, most notably, operative maneuvers were less frequently reported as the phase associated with the greatest pain. These findings suggest that the main determinant of procedural pain is not the intrauterine phase itself, but rather gaining access to the uterine cavity. This observation is consistent with the existing literature. Several studies have identified passage through the cervical canal as one of the most painful steps of outpatient hysteroscopy and have recognized cervical stenosis as one of the main factors associated with procedural pain and failure to complete the examination [12,19,30,31]. It is therefore plausible that anatomical conditions such as cervical stenosis, cervical tortuosity, or other difficulties in cervical access require additional maneuvers to reach the uterine cavity, thereby contributing to the greater pain observed in our patients.

Another interesting finding of our study is that patients who identified operative maneuvers as the most painful phase of the procedure reported relatively low overall pain scores (mean NRS 3.69 ± 2.53), lower than those reported by patients identifying cervical passage or uterine cavity distension as the most painful phase. The lowest pain scores overall were observed in patients reporting uniform pain or being unable to identify a specific painful phase (mean NRS 2.08 ± 2.19). This finding most likely reflects minimal overall procedural discomfort, with no single phase sufficiently salient to be identified as the most painful. By contrast, the highest pain scores were observed among those who identified cervical passage as the most painful step (mean NRS 6.44 ± 2.73; *p* < 0.001). Although this finding should be interpreted with caution, it suggests that, once uncomplicated access to the uterine cavity has been achieved, the operative phase itself is not necessarily the main determinant of procedural pain. This interpretation is further supported by the analysis of the “see-and-treat” procedures. Contrary to what might be expected, patients who underwent a successful see-and-treat procedure during the same session reported the lowest pain scores (mean NRS 3.91 ± 2.44), whereas the highest pain scores were observed in patients in whom the operative procedure could not be completed (mean NRS 6.47 ± 2.85). Patients who underwent diagnostic hysteroscopy alone reported intermediate pain scores (mean NRS 5.45 ± 2.96). Taken together, these findings further support the hypothesis that the principal determinant of procedural tolerability is not the operative intervention itself, but rather the ability to achieve uncomplicated access to the uterine cavity. Once the uterine cavity has been reached, even minimally invasive procedures performed using 5-Fr mechanical instruments appear to be generally well tolerated. This observation is fully consistent with the philosophy of the modern “see-and-treat” approach, which aims to integrate diagnosis and treatment within the same session while avoiding additional procedures, anesthesia, and hospitalization whenever clinically appropriate [1].

Postmenopausal women reported significantly higher pain scores than premenopausal women (mean NRS 5.90 ± 2.94 vs. 4.53 ± 3.03; *p* = 0.020). A plausible explanation for this finding may lie in the anatomical and functional changes associated with postmenopausal hypoestrogenism, including cervical atrophy, reduced elasticity, and an increased prevalence of cervical stenosis [19,31,32]. Cervical stenosis is more common in postmenopausal women and is associated with greater procedural pain and difficulty in accessing the uterine cavity. Therefore, difficult cervical access may contribute to the higher pain scores observed in postmenopausal patients.

The influence of uterine position on procedural pain remains controversial. Guraslan et al. identified uterine retroversion and excessive uterocervical angulation as independent predictors of painful office hysteroscopy, suggesting that marked uterine flexion may increase contact between the hysteroscope and the cervical canal, whereas retroverted or retroflexed uteri may require greater tissue compression during hysteroscope guidance, thereby making cervical passage more difficult and painful [33]. In contrast, we did not observe a significant association between the presence of a retroverted–retroflexed uterus and procedural pain (*p* = 0.426). Although the limited number of patients with RVF in our cohort (9/94) may have reduced the statistical power of this analysis, our findings suggest that uterine position alone may have only a limited influence on procedural pain when outpatient hysteroscopy is performed using a standardized vaginoscopic technique.

Obstetric history was not associated with procedural pain in our cohort. Neither a previous cesarean section (*p* = 0.671) nor a history of vaginal delivery (*p* = 0.551) significantly influenced the maximum pain experienced during outpatient hysteroscopy. Likewise, no significant differences were observed when patients were stratified according to obstetric history into nulliparous women, women with previous cesarean section only, previous vaginal delivery only, or both previous vaginal delivery and cesarean section (*p* = 0.941). These findings suggest that obstetric history alone may have only a limited influence on pain perception during outpatient hysteroscopy performed in a standardized vaginoscopic setting.

### Clinical Implications

From a clinical perspective, our findings suggest that the early identification of patients at increased risk of poor procedural tolerability may allow a more personalized hysteroscopic approach. The presence of potentially difficult cervical access, postmenopausal status, and high expected pain are factors that can be identified during the pre-procedural assessment and may support more comprehensive counseling, the adoption of strategies aimed at facilitating cervical access, or referral of more complex cases to experienced hysteroscopists.

At the same time, our findings indicate that these factors should not be considered a contraindication to outpatient hysteroscopy, which remains the reference standard for the diagnosis and treatment of benign intrauterine pathology. Indeed the high rates of procedural tolerability and willingness to undergo outpatient hysteroscopy again observed in our cohort confirm the high acceptability of outpatient hysteroscopy even in an unselected patient population and suggest that the goal should not be to identify patients who should be excluded from the outpatient setting, but rather to recognize those who may benefit from a more personalized approach aimed at optimizing procedural tolerability while maximizing the likelihood of successful completion of outpatient hysteroscopy. From the physician’s perspective, an incomplete outpatient hysteroscopy is more than a failed procedure; it represents a missed diagnostic and therapeutic opportunity.

## 5. Strengths and Limitations

The present study has several strengths. Its prospective design and the use of a highly standardized vaginoscopic hysteroscopic protocol minimized procedural variability and reduced potential technical confounders affecting pain assessment. In addition, the prospective collection of both patient-reported outcomes and physician-reported procedural findings through a structured questionnaire completed at predefined time points provided a comprehensive and multidimensional evaluation of the outpatient hysteroscopy experience. Finally, the simultaneous assessment of multiple clinical, psychological, and procedural variables allowed a broad evaluation of the factors potentially influencing pain perception and procedural tolerability within a standardized hysteroscopic setting.

Nevertheless, some limitations should be acknowledged. This was a single-center study with a relatively small sample size, which may limit the generalizability of our findings. Referral indications were not systematically collected as a predefined study variable. The questionnaire used for data collection was specifically developed for the purposes of this study and has not undergone formal psychometric validation. Likewise, pre-procedural anxiety was assessed using a simple Numeric Rating Scale rather than a validated anxiety questionnaire. Furthermore, although the definition of difficult cervical access was based on predefined criteria derived from the available literature, it has not yet been universally standardized and therefore requires external validation. Finally, although most procedures were performed by the same operator, thereby improving procedural standardization and reducing inter-operator variability, this may further limit the external generalizability of the results.

## 6. Conclusions

Office hysteroscopy remains a highly acceptable and well-tolerated procedure for the diagnosis and treatment of benign intrauterine pathology. In a standardized hysteroscopic setting, poor procedural tolerability appears to depend primarily on difficult cervical access rather than on the operative procedure itself, while patients’ expectations regarding pain may influence the hysteroscopic experience more than pre-procedural anxiety. Failure to gain access to the uterine cavity remains one of the greatest challenges in outpatient hysteroscopy, as it prevents completion of the examination and may preclude the opportunity to perform immediate treatment. Consequently, an incomplete office hysteroscopy should not be regarded merely as a failed procedure, but as a missed diagnostic and therapeutic opportunity. These findings support a personalized approach to outpatient hysteroscopy focused on the early identification of patients at increased risk of poor tolerability, with the aim of optimizing procedural completion and preserving the benefits of the outpatient see-and-treat approach.

## Figures and Tables

**Table 1:** **Baseline patient characteristics, procedural characteristics, and pain outcomes (*n* = 105).** Abbreviations: SD, standard deviation; IQR, interquartile range; NRS, Numeric Rating Scale; RVF, retroverted uterus. Note: Uterine position was assessable in 94 patients. In 11 patients, uterine position could not be determined because the diagnostic hysteroscopy was not completed.

Characteristic	Value
Age, years (mean ± SD)	50.6 ± 11.5
Age, years, median (IQR)	52 (44–59)
Menopause, *n* (%)	52 (49.5)
History of vaginal delivery, *n* (%)	53 (50.5)
Chronic pelvic pain, *n* (%)	13 (12.4)
Dyspareunia, *n* (%)	26 (24.8)
Previous cervical procedures, *n* (%)	7 (6.7)
Retroverted uterus (RVF), *n* (%)	9/94 (9.6)
Difficult cervical access, *n* (%)	37 (35.2)
Operative procedure performed, *n* (%)	57 (54.3)
Diagnostic procedure completed, *n* (%)	94 (89.5)
Vasovagal reaction, *n* (%)	4 (3.8)
Maximum pain score (NRS), mean ± SD	5.2 ± 3.0
Maximum pain score (NRS), median (IQR)	5 (2–8)
Post-procedural pain score (NRS), mean ± SD	2.2 ± 2.6
Post-procedural pain score (NRS), median (IQR)	1 (0–3)

**Table 2:** **Association between difficult cervical access and procedural pain. Note:** Severe pain (NRS ≥ 7): OR 8.04 (95% CI 3.25–19.87).

Variable	Difficult Cervical Access (*n* = 37)	No Difficult Cervical Access (*n* = 68)	*p*-Value
**Maximum pain NRS, mean ± SD**	7.35 ± 2.81	4.04 ± 2.51	<0.001
**Maximum pain NRS, median (IQR)**	8 (5–10)	4 (2–5.25)	<0.001
**Severe pain (NRS ≥ 7), *n* (%)**	25 (67.6%)	14 (20.6%)	<0.001

**Table 3:** **Maximum procedural pain according to the hysteroscopic phase perceived as the most painful.**

Phase of Maximum Pain	n (%)	Mean NRS ± SD	Median (IQR)
Cervical passage	57 (54.3)	6.44 ± 2.73	7 (5–9)
Uterine cavity distension	10 (9.5)	5.90 ± 2.81	5.5 (4–7.75)
Operative maneuvers	26 (24.8)	3.69 ± 2.53	3 (2–5)
Uniform pain/Unable to identify a specific phase	12 (11.4)	2.08 ± 2.19	1.5 (1–2.25)

**Table 4:** **Correlations between patient-reported factors and maximum procedural pain.**

Variable 1	Variable 2	Spearman’s ρ	*p*-Value	N
Expected pain (NRS)	Maximum pain (NRS)	0.304	0.00164	105
Anxiety (NRS)	Maximum pain (NRS)	0.132	0.1780	105

**Table 5:** **Association between menopausal status and maximum procedural pain.**

Variable	Menopause (*n* = 52)	No Menopause (*n* = 53)	*p*-Value
**Maximum pain NRS, mean ± SD**	5.90 ± 2.94	4.53 ± 3.03	0.020
**Maximum pain NRS, median (IQR)**	5.5 (3–8)	4.0 (2–7)	0.020

**Table 6:** **Association between operative procedure status and maximum pain during hysteroscopy. Note:** The 11 patients in whom diagnostic hysteroscopy could not be completed were excluded from this analysis; therefore, the denominator for Table 6 is 94 rather than 105.

Operative Procedure Status	*n*	Mean NRS ± SD	Median (IQR)
**Successful see-and-treat (S)**	57	3.91 ± 2.44	4 (2–5)
**Failed see-and-treat (N)**	17	6.47 ± 2.85	7 (4–9)
**Diagnostic hysteroscopy only (operative procedure not indicated)**	20	5.45 ± 2.96	5 (3.75–8)

**Table 7:** **Factors associated with failure to complete the diagnostic hysteroscopy. Note:** Standard unadjusted odds ratios were not estimable for these comparisons because of zero cells in the contingency tables.

	Incomplete Procedure	Completed Procedure
**Difficult cervix**	11	26
**No difficult cervix**	0	68
**Severe pain (NRS ≥ 7)**	11	28
**No severe pain (NRS < 7)**	0	66

## Data Availability

The data supporting the findings of this study are not publicly available due to privacy and ethical restrictions. Reasonable requests for access to portions of the dataset may be submitted to the corresponding author, together with a justification of the intended use, and will be considered on a case-by-case basis.

## Supplementary Materials

The following supporting information can be downloaded at https://www.mdpi.com/article/10.3390/jcm15166335/s1: File S1: Outpatient Hysteroscopy “See and Treat”—Questionnaire on Pain and Tolerability.
