# Supplementary material for: Clinical Factors Associated with Pain During Outpatient Hysteroscopy in a Standardized Vaginoscopic Setting: A Prospective Observational Study
Source: J Clin Med. 2026 Aug 16;15(16):6335. doi: 10.3390/jcm15166335 (PMC13513420; doi:10.3390/jcm15166335)
Supplement: Supplementary file 1 [file jcm-15-06335-s001.zip › jcm-4495446-supplementary.pdf]

OUTPATIENT HYSTEROSCOPY “SEE AND TREAT” – QUESTIONNAIRE ON PAIN AND TOLERABILITY

PATIENT INITIALS: ____________________________ **Date:** ____ / ____ / ______

SECTION A - BEFORE THE PROCEDURE

Age: ______ years

Are you menopausal?
☐ No ☐ Yes

If yes, do you use vaginal estrogen therapy (ovules/cream)?
☐ No ☐ Yes ____

Have you had vaginal deliveries?
☐ No ☐ Yes _____________

Have you undergone cervical procedures (LEEP/conization/cervical lacerations during delivery)?
☐ No ☐ Yes

Do you suffer or have you suffered from painful menstruation?
☐ No ☐ Mild ☐ Moderate ☐ Severe

If you had menstruations, how much pain do you usually experience?
(0 = no pain; 10 = maximum pain)

0 1 2 3 4 5 6 7 8 9 10

Do you have chronic pelvic pain (persistent or recurrent pain lasting more than 3 months)?
☐ No ☐ Yes

Have you experienced pain during sexual intercourse in recent months?
☐ No ☐ Yes

Have you previously undergone hysteroscopy or similar gynecological procedures?
☐ No ☐ Yes

If yes, do you remember them as painful?
☐ No ☐ Yes

How anxious do you feel at this moment?
(0 = not at all; 10 = very much)

0 1 2 3 4 5 6 7 8 9 10

How painful do you think the procedure will be?
(0 = not at all; 10 = very much)

0 1 2 3 4 5 6 7 8 9 10

SECTION B - AFTER THE PROCEDURE

Maximum pain experienced during the procedure
(0 = no pain; 10 = maximum pain)

0 1 2 3 4 5 6 7 8 9 10

At what moment did you experience the most pain?

☐ During passage through the cervix
☐ During distension / visualization of the uterine cavity
☐ During maneuvers (biopsy / lesion removal)
☐ I do not know / uniform pain

Current pain (now)
(0 = no pain; 10 = maximum pain)

0 1 2 3 4 5 6 7 8 9 10

If you had to undergo a similar procedure again, you would prefer:

☐ To repeat it in an outpatient setting in the same way
☐ To repeat it under sedation / not in an outpatient setting
☐ I do not know

Overall, the procedure was for you:

☐ Well tolerable
☐ Tolerable
☐ Difficult
☐ Intolerable

SECTION C - PHYSICIAN SECTION

Operator: ____________________________
Role: ☐ Senior ☐ Resident physician

Was diagnostic hysteroscopy completed?
☐ Yes ☐ No

If NO, main reason:

☐ Pain / intolerance
☐ Inability to overcome the cervical canal
☐ Bleeding
☐ Other: ____

Was at least one operative procedure performed in office?

☐ No ☐ Yes ☐ Not indicated

If YES, specify (multiple answers possible):

☐ Endometrial biopsy
☐ Targeted biopsy
☐ Endometrial polypectomy
☐ Cervical polypectomy
☐ Other: ____

Was the see-and-treat attempted but NOT completed due to pain/intolerance?

☐ No ☐ Yes

If YES, the procedure was interrupted:

☐ Before overcoming the cervical canal
☐ During cervical passage
☐ In the uterine cavity
☐ During operative maneuver

Difficult cervix (operational definition):

☐ No ☐ Yes

Uterine position:

☐ Anteverted–anteflexed (AVF)
☐ Retroverted–retroflexed (RVF)
☐ Other / not definable

Total procedure duration: ______ minutes

Immediate complications:

☐ No ☐ Yes → specify: ________________________________
